# Advances in Immunotherapy for Hepatocellular Carcinoma (HCC)

**DOI:** 10.3390/curroncol30110711

**Published:** 2023-11-07

**Authors:** Fuat Bicer, Catrina Kure, Anil A. Ozluk, Bassel F. El-Rayes, Mehmet Akce

**Affiliations:** 1 Division of Hematology Oncology, Department of Medicine, University of Cincinnati Medical Center, Cincinnati, OH 45267, USA; bicerft@ucmail.uc.edu; 2 Department of Medicine, Northside Hospital-Gwinnett, Lawrenceville, GA 30046, USA; catrina.kure@northside.com; 3 Division of Hematology Oncology, Department of Medicine, O’Neal Comprehensive Cancer Center, University of Alabama at Birmingham Heersink School of Medicine, Birmingham, AL 35233, USA; aozluk@uab.edu (A.A.O.); belrayes@uabmc.edu (B.F.E.-R.)

**Keywords:** HCC, immunotherapy, PD1, PDL1, TIGIT, LAG3, TIM3

## Abstract

Hepatocellular carcinoma (HCC) is the second most common cause of cancer-related deaths in the world. More than half of patients with HCC present with advanced stage, and highly active systemic therapies are crucial for improving outcomes. Immune checkpoint inhibitor (ICI)-based therapies have emerged as novel therapy options for advanced HCC. Only one third of patients achieve an objective response with ICI-based therapies due to primary resistance or acquired resistance. The liver tumor microenvironment is naturally immunosuppressive, and specific mutations in cell signaling pathways allow the tumor to evade the immune response. Next, gene sequencing of the tumor tissue or circulating tumor DNA may delineate resistance mechanisms to ICI-based therapy and provide a rationale for novel combination therapies. In this review, we discuss the results of key clinical trials that have led to approval of ICI-based therapy options in advanced HCC and summarize the ongoing clinical trials. We review resistance mechanisms to ICIs and discuss how immunotherapies may be optimized based on the emerging research of tumor biomarkers and genomic alterations.

## 1. Introduction

Primary liver cancer is the sixth most diagnosed cancer worldwide. Hepatocellular carcinoma (HCC) is the predominant subtype, accounting for close to 90% of cases [1,2]. There were varying incidences ranging from 6.3 per 100,000 in the United States to >50 per 100,000 in some countries in East Asia in 2020 [1]. It is the second leading cause of cancer-related death in men and sixth in women [3]. The gap in incidence rates is due to disparities in the prevalence of risk factors [3,4,5].

Hepatitis C virus (HCV) is the leading cause of HCC in Western Europe, North America, and Japan, while Hepatitis B virus (HBV) is the leading cause of HCC in Asia (besides Japan), South America, and Africa [6]. The prevalence of some modifiable risk factors is on the rise globally, including alcohol consumption, metabolic syndrome, and non-alcoholic fatty liver disease (NAFLD). One study found that >3 drinks per day was associated with a 16% increased risk of HCC [7]. For another risk factor, metabolic syndrome, one study found it was associated with an 81% increased risk of developing HCC, and that risk can be reduced by treating one of the many conditions attributed to metabolic syndrome such as insulin resistance, obesity, hypertension, and dyslipidemia [7].

Immune checkpoint inhibitor (ICI)-based therapies have emerged as novel therapy options in advanced HCC. Early approved ICI’s included targets for programmed cell death-1 (PD-1)/programmed cell death ligand-1 (PD-L1) in the form of antibodies. ICI’s have shown progressive improvements in overall survival outcomes compared to sorafenib (TKI). Combination agents targeting cytotoxic T lymphocyte-associated antigen 4 (CTLA-4) and (PD-1) have been approved as well, surpassing the ICI monotherapy overall survival rates. Approximately two thirds of patients do not achieve the objective response with ICI-based therapies due to primary or acquired resistance created by the tumor’s overwhelming immunosuppressive state [8].

There has been increasing attention on using small molecules to target PD-1/PD-L1. The motive behind these is the potential for toxicity, lower costs, and greater stability compared to ICI antibodies. None so far have been approved for clinical use, but there are preclinical studies and a few ongoing early-phase clinical trials, which will be discussed below [9].

Unfortunately, there are no specific biomarkers to predict who will respond or develop resistance to ICIs. However, recent preclinical studies and molecular analysis in key landmark trials have described the large role that specific genes alterations and baseline immune characteristics of the tumor may have on developing resistance and their potential as targets to overcome resistance. In this review, we outline current approved and developing therapies for advanced HCC, discuss the mechanisms of ICI resistance, and discuss potential solutions to overcome resistance.

### The Role of the Tumor Microenvironment in HCC

The tumor microenvironment (TME) of HCC is characterized by a heterogeneous group of immune cells, tumor cells, and cytokines in the setting of a chronically inflamed liver. Various mechanisms take place to permit tumor cell immune evasion and the development and progression of HCC.

A central theme to the success of the anti-tumor response is proper antigenicity. Tumor cells may display tumor-associated antigens (TAAs), which are peptides allowing the host immune system to recognize the tumor cells. Some examples of TAAs include alpha-fetoprotein (AFP) and glycpican-3 (GPC-3). They can be pre-existing or formed by the tumor as hepatocarcinogenesis occurs. A spontaneous immune response may occur during liver injury after recognition of the TAAs by the naturally occurring TAA-specific CD8+ T cells [10,11,12].

**Figure 1:**
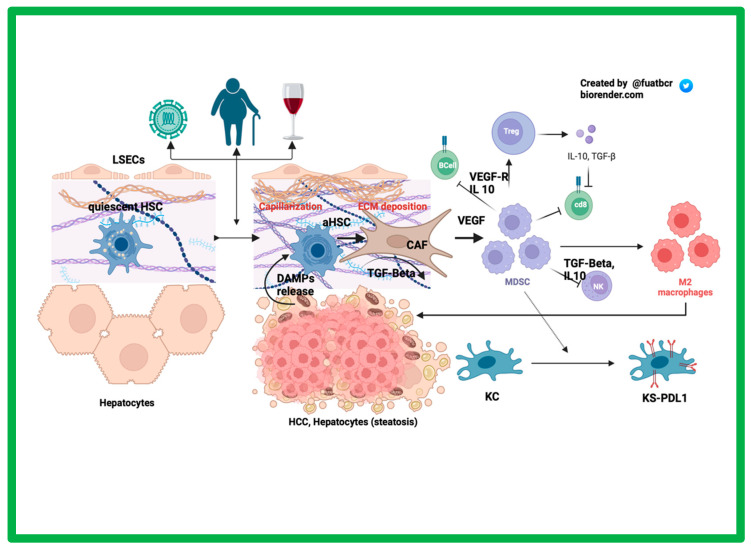
The role of the tumor microenvironment in HCC.

The liver endothelial sinusoidal cells (LESCs) are fenestrated cells lining the liver sinusoids important in inducing immune tolerance by acting as antigen-presenting cells (APCs) [13]. After chronic injury (Figure 1), LSECs undergo capillarization, meaning they lose their basement membrane and fenestrations, making it challenging for hepatocyte oxygenation. The hepatocytes in this hypoxic environment thereby undergo apoptosis and necrosis, releasing specific damage-associated molecular patterns (DAMPs), which can activate the typically quiescent hepatic stellate cells (HSCs), which then can transform to cancer-associated fibroblasts (CAFs) under the influence transforming growth factor-β (TGF-β) [14,15,16]. CAFs contribute to HCC progression by recruiting macrophages and converting them to an M2 macrophage (pro-tumor) phenotype and by upregulating T regulatory (Tregs) cells via secretion of vascular endothelial growth factor (VEGF) [17]. Surrounding the hypoxic hepatocytes, VEGF is released, which stimulates angiogenesis and Treg cell proliferation. Tregs are mostly known for their immunosuppressive effects, occurring via secretion of TGF-β1 and IL-10 in a chronically inflamed liver. Studies have found that VEGF receptor (VEGFR)-2 can increase Tregs presence in the tumor microenvironment [18]. Myeloid-derived suppressor cells (MDSCs) are also present in the TME and promote the expansion of Tregs. MDSCs interact with Kupffer cells and induce an immunosuppressive environment by upregulating their expression of PD-L1. MDSCs secrete IL-10 and VEGF to help recruit Tregs, which further contributes to the immune downregulation [19,20].

Conversely, CD8+ T cells provide an anti-tumor response. Earlier studies have shown that these cells are decreased in hepatocellular tumor tissue compared to that of the non-malignant tissue [21,22]. These cells also express an exhausted phenotype. One more recent study revealed that in HCC patients, there was more expression of the immune checkpoints on the CD8+ T cells in the malignant tissue compared to in the periphery [23].

Concerted efforts are underway to expand therapeutic armamentarium in HCC by inhibiting pro-tumor pathways and enhancing anti-tumoral immune cytotoxicity in several ongoing clinical trials [24].

## 2. Current First-Line Therapies for Advanced/Metastatic HCC

### 2.1. TKI-Based Therapies

#### 2.1.1. Sorafenib

**Figure 2:**
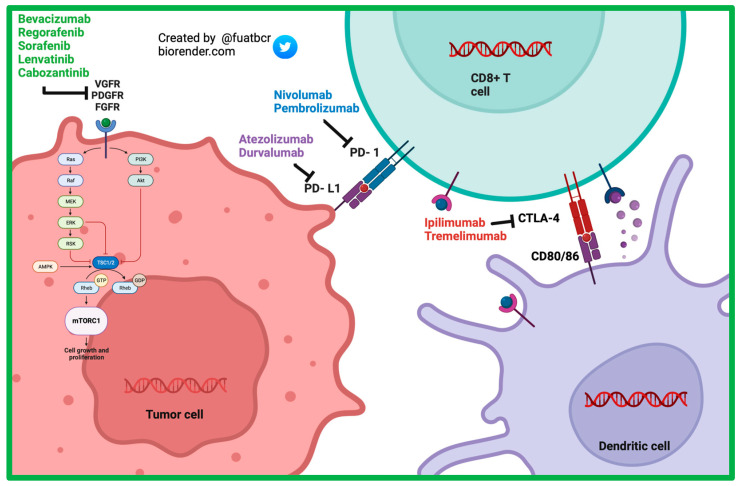
Mechanism of action of currently approved systemic therapy options in advanced HCC.

Sorafenib, a multi-kinase inhibitor, (Figure 2) was the first systemic therapy to gain FDA approval for the treatment of HCC. The landmark SHARP trial was a multicenter, randomized control phase III trial that included 602 patients assigned in a 1:1 ratio to receive 400 mg sorafenib or placebo. Eligible patients were Child–Pugh Class A and had no previous systemic therapy. Most patients had HCC caused by chronic HCV (56%) and alcohol consumption (52%), and chronic HBV (37%) closely followed [25]. Median OS was 10.7 months in the sorafenib group and 7.9 months in the placebo group (Hazard Ratio (HR) = 0.69; 95% confidence interval (CI) = 0.55 to 0.87; *p* < 0.001). The incidence of drug related serious adverse events (AEs) was 9.4–14.6% in the sorafenib group and 5.0–25% in the placebo group [26]. The subsequent Asia–Pacific study confirmed the findings of the SHARP trial, showing that the mOS was 6.5 months in the sorafenib arm and 4.2 months in the placebo arm (HR = 0.68; 95% CI = 0.50–0.93; *p* = 0.014). Inclusion and exclusion criteria were similar as well [25,27,28,29].

After sorafenib received FDA approval, multiple clinical trials with other drugs failed to improve survival when compared to sorafenib. These drugs include sunitinib, brivanib, linifanib, everolimus, and tivantinib [30,31,32,33,34,35].

#### 2.1.2. Lenvatinib

Lenvatinib is a multi-kinase inhibitor targeting VEGFR, fibroblast growth factor receptor (FGFR), platelet-derived growth factor receptor (PDGFR alpha), RET protooncogene (RET), and kit-protooncogene (KIT). In the REFLECT trial, a randomized phase III noninferiority trial, lenvatinib was found to be noninferior in overall survival compared to sorafenib (mOS 13.6 vs. 12.3 months, HR = 0.92; 95% CI = 0.79–1.06). The secondary endpoint of median progression-free survival (PFS) was 7.4 months in the lenvatinib group versus 3.7 months in the sorafenib group (HR = 0.66, 95% CI 0.79–1.06). Hypothyroidism, decreased appetite, and hypertension were more common in the lenvatinib group, and diarrhea and a hand–foot–skin reaction was less common the lenvatinib group. In 2018, the FDA approved lenvatinib for first-line treatment of patients with advanced HCC [36].

### 2.2. ICI-Based Therapies

#### 2.2.1. Atezolizumab and Bevacizumab

The combination of atezolizumab, an anti-PD-L1 antibody, and bevacizumab, an anti-VEGF antibody, has shown synergistic anticancer activity [37,38]. Bevacizumab blocks VEGF, enabling maturation of dendritic cells that would have otherwise been downregulated with VEGF activity. Blocking VEGF prevents upregulation of MDSCs, which in turn allows for proliferation of CD8+ T cells and suppression of Tregs. With this environment, there is adequate antigen presentation, but tumor cells still can inhibit cytotoxic activity of the T cells with PD-L1/PD-1 upregulation. With the addition of an anti PD-L1 antibody, the T cells are able to destroy cancer cells without inhibition [37].

The phase Ib clinical trial GO30140 was an open-label, multi-arm trial where one of the HCC cohorts (A) studied the safety and efficacy of atezolizumab plus bevacizumab, while the other cohort (F) studied atezolizumab plus bevacizumab versus atezolizumab. Both cohorts met their primary endpoints with statistical significance [39].

IMbrave150 is a phase III randomized trial in patients who had unresectable HCC, no prior history of systemic therapy, and well-compensated liver disease (Child–Pugh A (CPA)). Patients were randomized to atezolizumab + bevacizumab versus sorafenib in a 2:1 ratio. The mOS was 19.2 months in the combination arm and 13.4 in the control arm (HR = 0.66; 95% CI = 0.52–0.85; *p* < 0.001). Grade 3 or 4 treatment-related adverse events occurred in 43% of the atezolizumab + bevacizumab group and 46% of the sorafenib group [40].

A recent study analyzed the correlative baseline tumor samples from a group of patients enrolled in the GO30140 or IMbrave150 phase III trial and provided insight to potentially significant biomarkers (key correlative findings are summarized in Table 1) [41]. It was demonstrated that genes or immune markers associated with pre-existing immunity, including expression of PD-L1 mRNA and effector T cell (Teff), were correlated with higher response to atezolizumab + bevacizumab in both GO30140 cohort A and in the IMbrave150 trial. Validation with immunohistochemical analysis was not able to demonstrate a clinically significant relationship between PD-L1 levels and ORR but revealed higher rate of infiltration of CD8+ T cells in the responders in arm A of GO30140 cohort A and in the IMbrave150 trial. Additionally, the study revealed that patients in the IMbrave150 trial with a low ratio of Treg/Teff signatures had a statistically significant higher PFS and OS in the atezolizumab + bevacizumab combination therapy group compared to sorafenib.

The study was able to use the GO30140 cohort F correlative samples to study the benefits of the added bevacizumab. Immune subsets of CD8+ T cells, Treg cells, and macrophages were associated with outcomes with the addition of bevacizumab [41,42]. Effector T cell and myeloid gene signatures at baseline were associated with improved outcomes in the combination group. It was also shown that the expression of the VEGFR-2 gene (KDR) was decreased in the combination group compared to monotherapy in pre- and post-treatment tumor biopsies. In addition, 80% of the responders had a decrease in the Treg signature in the combination group compared to 33% of the responders in the atezolizumab group. Lastly, higher blood vessel density was associated with longer PFS in the combination group compared to monotherapy.

The study also investigated the impact of TMB on therapy outcomes using whole-genome sequencing (WES) on tumor-blood samples of patients in both trials. TMB was not associated with outcomes in the IMbrave150 group [41].

#### 2.2.2. Durvalumab + Tremelimumab

In a phase II trial involving patients with unresectable HCC, the combination of tremelimumab (anti-CTLA-4 antibody) plus durvalumab (a PD-L1 inhibitor) demonstrated promising clinical activity and safety [43]. Patients were randomly assigned to receive either 300 mg of tremelimumab for one dose plus 1500 mg of durvalumab every 4 weeks, 1500 mg of durvalumab every 4 weeks, 75 mg of tremelimumab every 4 weeks for a total of four doses plus 1500 mg of durvalumab every 4 weeks (a combination given the term T75 +D), or 400 mg of sorafenib twice a day.

The phase III HIMALAYA trial randomized previously untreated advanced HCC patients using the STRIDE (Single Tremelimumab Regular Interval Durvalumab) regimen, sorafenib, durvalumab, or 75 mg of tremelimumab every 4 weeks for a total of four doses plus 1500 mg of durvalumab every 4 weeks (T75 +D). Later, the T75 + D arm was closed based on the data from the phase II trial. The HIMALAYA trial demonstrated that the STRIDE regimen improved overall survival with mOS of 16.43 months versus 13.77 months for sorafenib (HR = 0.78; 96 CI = 0.65–0.92; *p* = 0.0035). The trial also demonstrated that durvalumab was noninferior to sorafenib. Based on the results of the HIMALAYA trial, the STRIDE regimen was approved for first-line therapy in advanced HCC [43,44].

An international, randomized phase III trial NCT03764293 studying camrelizumab plus rivoceranib, also known as apatinib (an anti-angiogenic), versus sorafenib recently revealed pivotal results in a front-line setting, with OS 22.1 months versus 15.2 months; HR = 0.62; 95% CI = 0.49–0.80; *p* < 0.0001. This study is now known as CARES-310, and a new drug application has just been submitted for the combination as a first-line treatment option [45].

## 3. Current Second-Line Therapies for Advanced/Metastatic HCC

### 3.1. ICI-Based Therapies

#### 3.1.1. Nivolumab

Checkpoint 040 trial was a multi-cohort, open-label clinical trial studying nivolumab as both monotherapy and in combination with ipilimumab in advanced HCC patients with prior sorafenib use and those who were sorafenib naive. Patients with Child–Pugh A were included in cohorts 1–3, which included a dose-escalation phase for safety and a dose-expansion phase to assess safety and clinical data for different doses of nivolumab monotherapy. The trial revealed ORRs of 15 and 20% in dose escalation and expansion phases, respectively. The FDA approved this drug for second-line treatment in HCC; however, it was later withdrawn [46]. In cohort 5, only patients with Child–Pugh B were included, and most patients were Child–Pugh B7 (76%). Patients were treated with nivolumab alone in this non-comparative study. The mOS of the Child–Pugh B group was 7.6 months (95% CI = 4.4–10.5). Patients who responded to nivolumab monotherapy in cohort 5 showed stable or improved liver function, evidenced by five of the six responders improving from Child–Pugh B to Child–Pugh A. All responders showed stable ALBI grades [47]. These findings signify the potential role that immunotherapy may have in reversing tumor-mediated decline in liver function.

The confirmatory Check-Mate-459 trial randomized previously untreated advanced HCC patients to nivolumab versus sorafenib but failed to improve OS. Therefore, the nivolumab approval based on the Checkpoint 040 trial was withdrawn [48].

#### 3.1.2. Pembrolizumab

Pembrolizumab originally received accelerated second-line approval for advanced HCC based on the findings of the Keynote-224 trial, which revealed an ORR of 17% per the RECIST v1.1 (95% CI = 11–26) and mOS of 12.9 months (95% CI 9.7–15.5) [49]. In the subsequent Keynote-240 trial assessing the safety and efficacy of pembrolizumab, mOS was 13.8 months in the pembrolizumab group and 10.6 months in the placebo group (HR = 0.78; 95% CI = 0.611–0.998; *p* = 0023), which did not meet the prespecified boundary of *p* = 0.0174 for OS [50].

Keynote-394, another phase III trial conducted in Asian patients with previously treated advanced HCC, revealed improvements in mOS and mPFS in those receiving pembrolizumab over best supportive care; mOS was 14.6 months for the pembrolizumab arm and 13.0 months for the placebo (HR = 0.70; 95% CI = 0.63–0.99; *p* = 0.0180). This trial is promising for the role of second-line ICIs for HCC in Asian patients [51,52].

#### 3.1.3. Nivolumab/Ipilimumab

In 2020, a nivolumab plus ipilimumab combination was approved for the treatment of patients with advanced HCC who were previously treated with sorafenib. This was based on results of Arm A of cohort 4 of the Checkmate 040 trial, where patients received nivolumab (1 mg/kg) with ipilimumab (3 mg/kg) every 3 weeks for a total of four doses followed by nivolumab (240 mg) every 2 weeks. The ORR was 32% (RECIST v1.1) while the median response duration was 17.5 months (4.6–30.5 months). A follow-up showed that the ORR continued to stay at 32% while the 24-month OS rate improved to 46% (95% CI = 32–59%) [46,53].

Currently approved TKI-based therapies in second-line and beyond are summarized in Table 2 with key findings of the landmark trials.

## 4. Combination Therapy of ICI with Anti-Angiogenic Therapy and TKI

The combination of immune checkpoint inhibitors with anti-angiogenic agents has changed HCC frontline therapy. Several other studies have taken advantage of the synergistic effect of an ICI with another novel agent [33].

The Keynote 524 trial was an open-label, phase Ib, multicenter, single-arm study where patients with unresectable HCC received lenvatinib and pembrolizumab. The primary objective was ORR via modified RECIST (mRECIST), RECIST version 1.1 (v1.1) per independent imaging review (IIR). One hundred out of the 104 patients did not receive any prior systemic therapy, and patients had BCLC stage B or C disease. The combination proved to have confirmed ORR of 46% (95% CI = 36–56) per mRECIST and 36% (95% CI = 26.6.0–46.2) per RECIST v1.1 [58].

Leap-002 was a phase III study that compared lenvatinib plus pembrolizumab versus lenvatinib plus placebo in previously untreated advanced HCC in a 1:1 ratio Child–Pugh Class A. The trial had co-primary endpoints of OS and PFS. The primary endpoints of OS and PFS in the combination of lenvatinib and pembrolizumab arm did not meet pre-specified statistical significance [59]. Although this trial failed to meet the pre-specified outcomes, it revealed significant survival data in both arms, with 21.2 months (95% CI = 19.0–23.6) in the lenvatinib plus pembrolizumab arm and 19.0 months (95% CI = 17.2–21.7) in the lenvatinib plus placebo arm.

The COSMIC-312 study is a phase III, multicenter, and open-label trial that studied the combination of cabozantinib and atezolizumab versus sorafenib. It randomized 837 patients with advanced HCC and no prior history of receiving systemic therapy to atezolizumab and cabozantinib versus sorafenib versus cabozantinib in a 2:1:1 ratio. The study had dual primary endpoints of PFS in the first 372 patients for the atezolizumab and cabozantinib versus sorafenib arm and OS for the atezolizumab and cabozantinib versus sorafenib in all patients. The primary endpoint of PFS was longer in the combination group (6.8 vs. 4.2 months; HR 0.63, 99% CI5.6–8.3, *p* = 0.0012), but there was no significant difference in the overall survival (15.4 vs. 15.5 months; HR 0.90, 96% CI 0.69–1.18, *p* = 0.44) between the groups [60]. Given the lack of improvement in survival, this combination therapy is unlikely to be adopted for first-line therapy in advanced HCC.

The ICI/anti-angiogenic combination trials so far have rendered impressive results as shown in the IMbrave 150 trial underscoring the unique synergistic activity of this combination approach. Although early phase trials with ICI/TKI combinations were promising, the survival benefit over single-agent TKI was not shown in larger trials. Several other studies involving anti-angiogenics or TKIs and ICIs are underway, as shown in Table 3, including tivozanib plus durvalumab (NCT03970616) and regorafenib plus tiselizumab (NCT04183088) [61,62,63].

## 5. Chimeric Antigen Receptor (CAR)-T Cell Therapy

CAR-T cell therapy involves modifying T cells genetically to express chimeric antigen receptors that enable precise targeting and elimination of tumor cells. Initially successful in blood cancers, CAR-T therapy is being explored to treat solid tumors, including HCC. A novel double-target CAR-T cell therapy has been developed, recognizing GPC3 (a protein upregulated in HCC) and inhibiting PD-1, demonstrating superior therapeutic effects on HCC compared to single-target CAR-T cells. These double-target CAR-T cells showed enhanced persistence, limited inhibitory receptor expression, and potent resistance against tumor cells [76]. In a phase I clinical trial focusing on Glypican-3 (GPC3), a significant HCC-associated antigen, as a promising target for heavily treated HCC patients CT017 CAR T cells co-expressing CAR-GPC3 and RUNX3 were engineered to induce CD8+ T-cell infiltration within the cancer microenvironment. The trial demonstrated a manageable safety profile, with all patients experiencing cytokine release syndrome (CRS) primarily at grades 2 and 3, which resolved post-treatment. Notably, one patient achieved a partial response, and two had stable disease, resulting in a 16.7% objective response rate and a 50% disease control rate [76,77,78,79].

## 6. Small-Molecule Inhibitors

Small molecules blocking PD-1/PD-L1 pathway may have shorter half-life, increased tissue penetration, oral bioavailability, increased anti-tumor activity and lower toxicity compared to monoclonal antibodies [80]. However, only a few have made it to clinical trials and HCC specific preclinical trials are still lacking.

The molecule CA-170, the first oral small molecule to target PD-L1, demonstrated acceptable safety in a phase I study in Hodgkin lymphoma and solid tumors known to express PD-1, including but not limited to renal cell carcinoma (RCC), melanoma, and non-small cell lung cancer [81,82]. The phase II study reported an ORR of 30% in the Hodgkin lymphoma group. Updated results of the phase II study reported a clinical benefit rate (CBR) of 75% in the no-squamous NSCLC. Currently, this agent is being studied in NSNSCLC in a phase IIb/3 clinical trial [83,84].

Most recently, a preclinical study of CCX559, a PD-L1 small-molecule inhibitor, was shown to achieve reversible PD-L1 internalization, activation of T cells, and anti-tumor activity in murine models [85,86]. An ongoing phase I study with single-agent CCX559 in solid tumors reported on-target pharmacokinetic effects suggesting PD-L1 inhibition [87]. Tubeimoside-1 (TBM-1) is a small molecule derived from the herb Bolbostemma paniculatum [88]. Liu at al. demonstrated that this molecule can induce lysosomal degradation of PD-L1 in cancer cells via mTOR inactivation [89]. A recent study reported that diminishing mitochondria oxidative phosphorylation (OXPHOS) can inhibit PD-1 expression. LND was already known to disrupt OXPHOS at high doses but did not have mitochondria-specific targeting abilities. Therefore, the study created a small molecule, IRLND@Alb, using a triphenylphosphonium (TPP+), a mitochondrial target. It is also attached to albumin to promote tumor accumulation through improved permeability. It was shown that this molecule was able to downregulate PD-L1 expression compared to the control (*p* < 0.05) [90]. Table 4 and Table 5 summarize the preclinical and clinical studies with small-molecule inhibitors [86]. At this moment, there are no small molecules against CTLA-4. The one study that looked at a B7-1 blockade to prevent CTLA-4 binding showed no inhibition.

## 7. Resistance Mechanisms to ICIs in HCC and Possible Solutions

Several clinical trials with ICI-based therapies have shown its capabilities in warding off tumor cells; however, many patients either do not achieve objective response or develop resistance to immune checkpoint inhibitors. The mechanisms of resistance can be categorized broadly into internal and external (Table 6). An internal resistance mechanism is one caused by the tumor itself, and an external resistance mechanism is caused by the tumor’s interaction with other cells in the TME.

### 7.1. Internal Resistance

#### 7.1.1. TMB

Tumor mutational burden (TMB) refers to the number of mutations per megabase in a tumor’s genome. Neoantigens are antigens derived from tumor cells or from the self and contribute the total TMB. It has been studied that when a tumor has a high TMB, many neoantigens are processed and presented by APCs to neoantigen-specific T cells, and the tumor becomes more immunogenic. Research has shown that a high TMB has been associated with a better ICI response in several solid tumor types [93,94,95]. However, this association is not consistent in HCC [96].

#### 7.1.2. Gene Signatures and Biomarkers

Correlation of gene signatures and response to therapy is an area of intense research in patients receiving ICI therapy [93,95]. Molecular classification of HCC through whole-exome sequencing has revealed an association between CTNNB1 mutation and immune evasion [41]. CTNNB1 mutation leads to an overly active Wnt/beta-catenin pathway [97]. Subsequently, this leads to a decrease in CD8+ T cells and an increase in Treg cells in the tumor environment [98]. Harding et al. noted that in the ten out of twenty-seven HCC patients with either Barcelona Clinic Liver Cancer (BCLC) Stages B or C treated with immunotherapy, seven had the CTNNB1 mutation while three had another mutation leading to an active Wnt pathway. All 10 patients were refractory to immunotherapy [99].

Zhu et al. also studied this mutation in tissue samples from the IMbrave 150 phase III trial and found no prognostic value of the CTNNB1 mutation status in the atezolizumab + bevacizumab group [41]. However, patients in the sorafenib group with the mutation had a longer PFS and OS. Prior clinical data noted that sorafenib has potential to decrease Wnt pathway signaling [100]. The study ultimately concluded that the similarity in survival benefit between the wild-type and mutant groups in the IMbrave150 study indicates that the addition of anti-angiogenics such as bevacizumab could help overcome the Wnt pathway-induced resistance to atezolizumab. Additionally, several other preclinical studies have shown that VEGFA is decreased after B catenin knockdown in HCC [101,102].

In the same study by Zhu et. al, it was noted that in the phase Ib study (GO30140), a high expression of the VEGF receptor 2 (KDR gene) was associated with greater PFS in the combination group compared to the group who received atezolizumab monotherapy. This validates that bevacizumab aids synergistically in the anti-tumor response by also inhibiting angiogenesis [41].

Several biomarkers including the CD274 gene (PD-L1 mRNA) and genes encoding effector T cells were associated with greater outcomes at higher expressions compared to lower expressions in the combination group in both the IMbrave 150 study and the GO30140 study (cohort A) [41].

Specific biomarkers can be isolated from tumor cells via next-generation sequencing (NGS). Tumor tissue biopsy has been a conventional method, but it does have limitations with inaccessible or smaller tumors. Cell tumor DNA (ctDNA) is DNA derived from tumor cells. ctDNA is released into the blood after apoptosis and has emerged as a non-invasive way of analyzing tumor biomarkers [103].

**Table 6:** Immunotherapy resistance mechanisms [104,105].

Internal Mechanisms	External Mechanisms
1. Gene mutation: [41,100] a. CTNNB1 mutation: [106] ↑ Wnt/beta-catenin pathway associated with ↑ T reg cells, ↓ CD8+ T cells	1. ↑ Immune checkpoints: [105] a. PD-1 b. PD-L1 c. CTLA-4 d. LAG-3 e. TIM-3 f. TIGIT
2. Gene expression variation: [107] a. ↓ CD274 gene expression (PD-L1 mRNA) associated with ↓ benefit from atezolizumab + bevacizumab	2. ↓ CD8+ T cells: [108] a. ↑ Pro-tumor state
3. ↓ Tumor mutational burden *: [105]	3. ↑ T reg cells: [108] a. ↑ Pro-tumor state

* Association of high TMB and ICI resistance has inconsistent data in the literature for HCC.

### 7.2. External Resistance

One external resistance mechanism is the development of immune checkpoint molecules by tumor cells. When presented with an antigen, the tumor cell can upregulate checkpoint molecules to evade the immune response. These molecules include PD-L1, PD-1 and CTLA-4, lymphocyte activation gene-3 (LAG-3), T-cell immunoglobulin and mucin domain (TIM-3), and TIGIT [89]. Studies have shown that the increased expression of TIM-3 in many cancers, including liver cancer, is associated with a poorer prognosis [109]. It was observed that persistent exposure to an anti PD-1 antibody upregulated TIM-3 expression in a tumor-bearing lung in mouse models, which supports the idea that a TIM-3/PD-1 blockade may have great therapeutic potential [110,111,112].

Another external resistance mechanism is the decreased infiltration of pro-tumor cells. The immunosuppressive cellular components of the liver TME includes Treg cells and CD8+ T cells [113]. The activation and proliferation of Treg cells inhibit CD8+ T cells, thus allowing tumor growth and progression [21]. Combatting overactive Treg function is a field that requires further studies in advanced HCC, but recent findings looking at the CTNNB1 gene have unmasked significant correlations between these CD8+ T cells and response to ICIs. External resistance mechanisms may overlap with internal ones, as gene alterations may alter the immune phenotype and cells involved [106].

## 8. Immunotherapy Combined with Locoregional Therapies

The combination of immunotherapy and liver-directed therapy is a promising approach that integrates two distinct strategies to improve outcomes in HCC. Immunotherapy utilizes the body’s immune system to recognize and attack cancer cells, while liver-directed therapy directly targets and treats tumors within the liver. Combining these approaches can potentially synergize their effects and improve overall treatment outcomes [114]. Radiofrequency ablation (RFA), microwave ablation (MWA), transarterial chemoembolization (TACE), stereotactic body radiation therapy (SBRT), and yttrium-90 (Y-90) radioembolization are all local therapies that aim to shrink and control tumors within the liver. This can make the remaining cancer cells more susceptible to the immune system, which is then boosted by immunotherapy. Tumor antigens released due to tumor destruction by liver-directed therapy can synergize with ICIs and achieve better anti-tumor response. Overall, combining immunotherapy with liver-directed therapy is a promising approach that capitalizes on their complementary mechanisms of action. This can lead to enhanced tumor control, systemic immune activation, and potential synergistic effects for a more effective and comprehensive treatment of liver cancers [28,115,116].

Clinical trials have explored the incorporation of RFA with either molecular targeting agents or immunotherapy. In a comparative analysis involving patients with primary HCC, a noteworthy extension in progression-free survival (PFS) was observed in the RFA combined with cellular immunotherapy (CIT) group (44 months vs. 30 months, *p* = 0.025) [117]. Additionally, a randomized trial comparing combined RFA with [(131)I] metuximab versus RFA alone in patients with BCLC 0-B HCC showcased a superior anti-recurrence advantage in the combined treatment cohort (median overall tumor recurrence of 17 months vs. 10 months, *p* = 0.046) [118]. A phase III randomized controlled trial revealed that HCC patients who underwent curative treatments (surgery, RFA, or percutaneous ethanol injection) and received adjuvant immunotherapy with activated cytokine-induced killer (CIK) cells experienced prolonged RFS and OS compared to those without adjuvant immunotherapy (median RFS of 37 months vs. 19 months, *p* < 0.001; median OS of 67 months vs. 41 months, *p* < 0.001) [119]. Subsequently, a retrospective analysis of the same patient cohort in Korea demonstrated that adjuvant immunotherapy following curative treatments (surgery or RFA) led to significantly extended RFS (median RFS of 45 months vs. 28 months, *p* < 0.001) [120]. Another retrospective study involving patients with established recurrent HCC who underwent either RFA alone or RFA coupled with anti-PD-1 therapy exhibited a notably higher 1-year recurrence-free survival rate in the group receiving anti-PD-1 plus RFA (32.5% vs. 10.0%, *p* = 0.008) [121].

Several ongoing clinical trials combining liver-directed therapy with ICIs hold promise in advancing the treatment of HCC. One such trial, NCT03753659, is a multicenter, single-arm, prospective, open-label phase II study examining the clinical efficacy of peri-interventional treatment using the anti-PD-1 antibody pembrolizumab in HCC patients eligible for local ablation via various methods such as RFA, MWA, brachytherapy, or a combination of TACE with RFA, MWA, or brachytherapy [122]. Another trial, NCT04663035, compares CT-guided thermal ablation plus tislelizumab versus ablation alone for intrahepatic recurrent early-stage hepatocellular carcinoma through a randomized controlled phase II clinical trial [123]. In a different study, the NCT04727307 trial aims to address the high intrahepatic distant recurrence rate and investigate adjuvant/neoadjuvant strategies targeting tumor growth and metastatic escape in the context of percutaneous thermal ablation for small HCC [124]. Additionally, NCT04652440, a phase II, single-arm, single-center study, is assessing the safety and tolerability of combining radiofrequency or microwave ablation with a PD-1 monoclonal antibody in HCC patients [125]. The study also aims to evaluate the efficacy of this combination and its effect on immune function and hepatitis virus infection status in patients with HCC. The study is divided into two stages, with the first stage focusing on dose-limited toxicity observation in six patients. If dose-limited toxicity is observed in fewer than two patients, the second stage will enroll an additional twenty-four patients for further evaluation [126].

## 9. Potential Solutions to Overcome Resistance to Immune Checkpoint Inhibitors in HCC via Targeting Other Checkpoint Molecules

Preclinical studies and analysis of clinical samples from the recently completed clinical trials in HCC shed light on some of the possible resistance mechanisms to ICI-based therapies. Targeting other immune checkpoints such as TIM-3, LAG-3, and T-cell immune receptors with immunoglobulin and ITIM domains (TIGITs) in combination with anti-PD-1/PD-L1/CTLA-4 pathways, or other relevant targets such as VEGF and VEGFR, are potential solutions. Table 7 outlines the mentioned targets.

### 9.1. TIM-3

Targeting PD-1 and TIM-3 simultaneously is an emerging concept. Two models of lung cancer harboring oncogenes KRAS or EGFR oncogenes were studied during their treatment with anti-PD-1 antibodies [110]. They were treated until they developed resistance, which was defined as initial response to therapy with a subsequent increase in the tumor size >120% of the initial size of the tumor. In the treated models who developed resistance, an upregulation of the TIM-3 antibody was found and confirmed via flow cytometry. To assess if blocking TIM-3 would have a therapeutic effect at time of resistance, an anti-TIM-3 antibody was administered. The cohort treated with anti-TIM-3 antibody demonstrated a greater median survival of 11.9 weeks compared to the group treated with the anti-PD-1 antibody alone (*p* = 0.0008). To see if these results mirror the patterns of resistance in patients with lung cancer, two patients who were treated with an anti-PD-1 antibody were analyzed, and it was found that TIM-3 expression was higher in the specimens from the patients who developed resistance to anti-PD-1 therapy compared to the samples from patients who did not receive anti-PD-1 therapy. This study suggests that there may be an adaptive resistance mechanism to anti-PD-1 antibody via the upregulation of TIM-3, and that targeting this may augment the anti-PD-1 response.

Although this study focused on markers in lung adenocarcinoma, an immunohistochemical study was performed that analyzed the expression of PD-1 and TIM-3 in HBV-associated HCC compared to HBV-induced liver cirrhosis, and it found elevated expression of TIM-3 in the HCC tissues over the cirrhosis tissues (*p* < 0.001) [127]. It was also reported that the expression levels positively correlated with the HCC grades, and those patients with grades 3 and 4 had significantly higher expression levels of both markers than those patients with lower grades.

One of the first clinical trials studying a combination of TIM-3 blockade and PD-1 blockade in HCC recently shared their interim results. In this phase II trial, 42 patients with BCLC stage B or C HCC are enrolled to receive cobolimab (anti-tim-3 antibody) 300 mg and dostarlimab (anti-PD-1 antibody) 500 mg on day 1 of each 21-day cycle for a maximum of 2 years, or until there is treatment failure. The primary objective is ORR. On 1 September 2022, 16 patients, with a median age of 68 years, had been enrolled. Only one patient had complete response, five patients had partial response (ORR of 46%), three patients had stable disease (23%), and four patients had disease progression (31%). There was only one grade 4 treatment-related adverse event of neutropenia, and the rest of the events were grade 1 and 2 and included pruritis, rash, and fatigue [68]. A phase I/II clinical trial investigating the TIM-3 antibody (BGB-A425) + tislelizumab (an anti-PD-1 antibody) in patients with previously treated locally advanced solid tumors is underway [128].

### 9.2. LAG-3

Analysis of tumor-infiltrating lymphocytes in HCC revealed that LAG-3 expression was upregulated in HBV-specific CD8+ T cells compared to the CD8+ T cells in the peripheral blood [129]. The study also demonstrated a correlation between LAG-3 and the amount of dysfunctional CD8+ T cells in the HBV-specific CD8+ T cells, further supporting the relationship between the two.

Initially studied alone, LAG-3 has recently been studied alongside PD-1 [130,131]. The first preclinical studies came from those studying ovarian and colorectal cancer. Woo et al. hypothesized that these immune checkpoint molecules can work synergistically to reduce tumor growth. They studied the dual blockade of LAG-3/PD-1 and tumor response in Sa1N fibrosarcoma and MC38-colorectal adenocarcinoma-inoculated mice. Tumor resolution occurred in 70–80% of the Sa1N fibrosarcoma and MC38-colorectal adenocarcinoma-inoculated mice, respectively. Of the two groups, much less tumor resolution occurred (0–40%) in the mice treated with anti-PD-1 or anti-LAG-3 blockade [132]. Another preclinical study examined mechanisms of enhanced anti-tumor immunity with a dual blockade of LAG-3 and PD-1 in an ovarian tumor murine model [133]. It revealed that there was an increase in CD8+ T cell and CD4+ T cell infiltration in the tumor environment along with a decreased number of Treg cells after blockade. It also confirmed that the influx of the CD8+ T cells were not exhausted T cells by making note of the increased amount of IFN-γ and TNF-α cytokines, indicating that they were active.

Guo et al. utilized multiplex immunofluorescence to examine the distribution of LAG-3, PD-L1, and CD8+ T cell expression in HCC tissue after hepatectomy compared to matched non-tumor tissue in patients. They concluded that expression of LAG-3 was an independent predictor of worse overall survival, which is similar to findings of other studies, which found that LAG-3 predicted worse overall survival in melanoma and non-small cell lung cancer [134,135,136].

In another HCC-specific study, Guo et al. demonstrated that patients with a high LAG-3 level prior to TACE therapy were correlated with worse disease outcome. Patients with elevated LAG-3 and PD-L1 levels had poorer overall survival compared to those with only PD-L1 or LAG-3 elevated in the same study [137].

Conversely, a study in 2023 by Wei et al. revealed that PDCD-1 (gene of PD-1) and LAG-3 polymorphisms did not influence the risk of HCC. However, a limitation to this study could be that the samples were obtained from the peripheral blood, compared to the other studies mentioned, which included samples from HCC tissue [138].

Ongoing clinical trials with dual LAG-3 and PD-1 blockade in HCC have recently been launched. RELATIVITY-073 is an ongoing phase II trial where patients with advanced HCC who progressed on TKI and who are naïve to immunotherapy are being randomized in a 2:1:2 ratio to either nivolumab (arm A) or one of two regimens of relatinib + nivolumab (arms B and C) [139]. Patients must have proven LAG-3 expression and be Child–Pugh class A. The primary endpoint is ORR.

RELATIVITITY-106 is a novel phase I/II trial studying the combination of nivolumab + relatinib + bevacizumab compared to nivolumab + bevacizumab in treatment-naïve, advanced HCC patients. The primary endpoints are PFS and incidence of dose-limiting toxicities [75].

Initial results from a phase I/II dose escalation and expansion trial studying tebotelimab, a PD-1/LAG-3 bispecific antibody in advanced HCC patients who had failed prior immunotherapy, revealed that tebotelimab had a tolerable safety profile [140,141,142].

### 9.3. TIGIT

A T-cell immune receptor with immunoglobulin and ITIM domains (TIGIT) is a co-inhibitory molecule expressed on activated T cells (Treg, CD8+ T cells, and CD4+ T cells), B cells, and NK cells. Chiu et al. studied the mechanisms of mice liver tumor resistance via mass cytometry. In this study, the anti-PD-1 antibody not only did not inhibit tumor growth, but it also led to the mice harboring many more T cells expressing PD-1, LAG-3, and TIGIT compared to the non-treatment mice. After injection of a combination of the anti-PD-1 antibody and anti-TIGIT antibody, there was evidence of reduced tumor growth, increased overall survival, and more expression of CTLs [143].

Ge et al. studied the role of blockade of these proteins in human HCC tissue and found that co-blockade of TIGIT and PD-1 resulted in an enhanced proliferation of CD8+ T in nivolumab non-responders [144]. The study also noted that the CD8+ T cells with high expression of PD-1 with co-expression of TIGIT also expressed the other inhibitory receptors such as TIM-3 and LAG-3. They concluded that this specific subset includes the most exhaustive CD8+ T cells.

In addition, other studies in HCC tissue found a negative correlation between the levels of expression of TIGIT and the degree of tumor progression [145,146]. As a TIGIT blockade seems to only affect exhausted T cells and is more specific on where and when it is expressed, there is potential that this blockade may produce fewer treatment-related adverse events than other checkpoint blockades [147].

The MORPHEUS-liver study is a novel phase Ib/II study investigating the combination of an anti-TIGIT therapy and tiragolumab, along with bevacizumab + atezolizumab versus the control arm of atezolizumab + bevacizumab in previously untreated patients with unresectable HCC. At the median follow-up of 14.0 months in the experimental group and 11.8 months in the control group, ORR was 43.5% in the experimental group and 11.1% in the control group, and no new safety-related concerns arose. This trial builds on the data of the preclinical studies and identifies a potentially new first-line agent for unresectable HCC if confirmed in larger trials [148]. The phase III study, IMbrave152/SKYSCRAPER-14, will be the next trial to study the efficacy and safety of this novel combination and is expected to start in July 2023 [149].

**Table 7:** Emerging checkpoint inhibitors in preclinical and clinical trials.

TIM-3 Preclinical Studies	
Study	Findings
Anti-TIM-3 blockade after PD-1 failure in lung cancer mice models [110]	OS: 11.9 weeks in TIM-3 blockade after PD-1 failure versus 5.0 weeks in PD-1 blockade monotherapy (*p* = 0.0008) in mice
PD-1 and TIM-3 expression in HBV-associated HCC versus cirrhosis [127]	Greater PD-1 expression in tumor tissue compared to surrounding cirrhosis tissue (*p* < 0.001) Greater TIM-3 expression in tumor tissue compared to cirrhosis tissue (*p* < 0.001)
LAG-3 Preclinical Studies	
Mechanisms of enhanced anti-tumor immunity with dual blockade of LAG-3 and PD-1 in an ovarian murine tumor model [133]	Increase in CD8+ T cells and decrease in Treg cells after blockade CD8+ T cells were not exhausted
Tumor response with LAG-3 and PD-1 blockade in Sa1N fibrosarcoma and MC38-colorectal adenocarcinoma [132]	Combination: tumor resolution (% population): Sa1N fibrosarcoma: 70% MC38-colorectal adenocarcinoma: 80% Monotherapy: tumor resolution PD-1 and LAG-3 monotherapy: 0–40%
Outcome of PD-L1 and LAG-3 expression in HCC [137]	Patients with high LAG-3 and PD-1 had poorer overall survival compared to elevation of only LAG-3 or PD-1
TIGIT Preclinical Studies	
Mechanisms of resistance of anti-PD-1 blockade in mice liver tumor and effects of PD-1 and TIGIT blockade in mice liver tumor [143]	Anti-PD-1 blockade led to the mice harboring many more T cells expressing PD-1, LAG-3, and TIGIT compared to the non-treatment mice After anti-PD-1 anti-TIGIT blockade, there was evidence of reduced tumor growth, increased overall survival, and more expression of CD8+ T cells
Effect of TIGIT and PD-1 blockade on CD8+ T cells; CD8+ T cells effect on antibody response [144]	Dual blockade enhanced proliferation of CD8+ T cells compared to single blockade (*p* < 0.05) Tumors with CD8+ T cell depletion did not show response to anti-TIGIT and PD-L1 blockade
TIGIT expression of T cells in healthy donors compared to those with chronic HBV infection [147]	TIGIT expression was highest for effector T cells in chronic HBV infection compared to healthy donors

## 10. Conclusions and Future Direction

Advanced HCC can be treated with several FDA-approved agents including ICI-based therapies with or without anti-angiogenics and TKIs. Two thirds of patients do not respond to ICI-based therapies. Identification of biomarkers is an urgent unmet need. Recent correlative analysis of baseline tumor samples from a group of patients from GO30140 or IMbrave150 shed light on potential biomarkers. Robust ongoing efforts in CAR- T cell therapy, targeting other checkpoint molecules such as TIM-3, LAG-3, and TIGIT, may expand ICI-based therapeutic options for HCC. Depending on further research, small-molecule inhibitors targeting the PD-1/PD-L1 pathway may create an alternative with more oral bioavailability, anti-tumor activity, and less toxicity.

## Figures and Tables

**Table 1:** Summary of molecular correlative analysis results in GO30140 and IMbrave 150 trials [41].

GO30140 Cohort A: Atezolizumab + Bevacizumab		
Gene Alterations or Immune Signatures	Immune Cell Types	TMB
Gene alterations or immune signatures associated with greater response: CD274 (PD-L1 mRNA): high expression associated with longer PFS compared to those with low expression (*p* = 0.0011). Teff: high expression associated with longer PFS in combination compared to those with low expression (0.0035).	Higher density of CD8+ T associated with better response (*p* = 0.007).	Greater ORR in TMB-high group (56%) compared to TMB-low group (35%).
**Phase III IMbrave 150 Trial: Atezolizumab + Bevacizumab vs. Sorafenib**		
**Gene Alterations or Immune Signatures**	**Immune Cell Types**	**TMB**
Gene alterations or signatures associated with greater response: CD274 (PD-L1 mRNA): high expression associated with longer PFS in combination group versus sorafenib (*p* = 0.015), as well as greater OS (0.002) Teff: high expression associated with longer PFS in combination group versus sorafenib (*p* = 0.047), as well as greater OS (0.0002)	Higher density of intra-tumoral CD8+ T cells showed longer PFS (0.053) and OS (0.001) Low ratio of Treg/Teff signatures had higher PFS and OS compared to sorafenib Higher density of CD8+ T cells associated with longer OS and PFS compared with sorafenib	No associations of TMB with outcome
**GO30140 Cohort F: Atezolizumab + Bevacizumab vs. Atezolizumab**		
**Gene Alterations or Immune Signatures**	**Blood Vessel Density**	
Genes or signatures associated with greater response: Myeloid inflammation: high expression associated with greater PFS (*p* = 0.036 versus monotherapy Gene signatures of Teff: high expression associated with greater PFS (*p* = 0.034 versus monotherapy KDR (VEGF receptor 2): high expression associated with greater PFS in combination group compared to monotherapy (*p* = 0.011)	High vessel density in baseline tumors associated with longer PFS in combination group compared to monotherapy (*p* = 0.0018)	

**Table 2:** Results from clinical trials from approved systemic therapies in advanced HCC.

Clinical Trials in HCC	Phase	Line of Therapy	Arms	Primary Outcome(s)	Median OS (Months)	ORR (%)	Year Approved
Multikinase inhibitors and monoclonal antibody against VEGFR2							
SHARP [26]	III	First	Sorafenib (S) Placebo (P)	OS	S: 10.7 P: 7.9 (HR = 0.69; 95% confidence interval (CI) = 0.55–0.87; *p* < 0.001)	S: 43 P: 32 *p* = 0.002	2007
RESORCE [54]	III	Second (post-SOR)	Regorafenib (R) Placebo	OS	R: 10.6 P: 7.8 (HR = 0.63; 95% CI = 0.50–0.79; *p* < 0.0001)	R:11 P: 4 *p* = 0.0047	2017
REFLECT [36]	III	First	Lenvatinib (L), Sorafenib	OS	L: 13.6 S: 12.3 (HR = 0.92; 95% CI = 0.79–1.06)	L: 18.8 S: 6.5 *p* < 0.0001	2018
CELESTIAL [55]	III	Second (post-SOR or other)	Cabozantinib (C) Placebo	OS	C: 10.2 P: 8.0 (HR = 0.76; *p* < 0.005)	C:4 P < 1 *p* = 0.009	2019
REACH-2 [56]	III	Second	Ramucirumab (Ra), Placebo (AFP ≥ 400 ng/mL)	OS	Ra: 8.5 P: 7.3 (HR = 0.71; *p* < 0.019)	R:5 P:1 *p* = 0·1697	2019
Immunotherapy (monotherapy)							
Keynote-224 [49]	II	Second	Pembrolizumab (Pem) (post-SOR)	ORR	Pem: 12.9 months (95% CI = 9.7–15.5)	17 (95% CI = 11–26)	2018
Checkmate 040 (cohorts 1–3 in dose expansion phase) [46]	I/II	Second	Nivolumab (N) (post-SOR)	ORR	6 months:83% 9 months:74%	20 (CI = 15–26)	2017
MKI with ICI							
IMbrave150 (2020) [57]	III	First	Atezolizumab + Bevacizumab (AB), Sorafenib		AB: 19.2 S: 13.4	A + B:30 S:11	2020
Dual checkpoint inhibitors							
Checkmate 040 (cohort 4)	I/II	Second	Nivolumab + ipilimumab	ORR	Arm A: 22.8 months (95% CI, 9.4-not reached) Arm B: 12.5 months (95% CI, 7.6–16.4) Arm C: 12.7 months (95% CI, 7.4–33.0)	ARM A: 32 (95 = CI 20–47) ARM B: 27 (95% CI = 15–41) ARM C: 29 (95% CI = 29 (17–43)	2020
HIMALAYA [44]	III	First	Durvalumab + Tremelimumab (STRIDE), Durvalumab (D), Sorafenib	OS	STRIDE: 16.4 S: 13.8 (HR = 0.78; 96% CI = 0.65–0.92; *p* = 0.0035) Durvalumab did not demonstrate superiority to sorafenib (*p* = 0.0674)	STRIDE:20.1 D: 17 S: 5.1	2022

**Table 3:** Ongoing clinical trials of ICI-based approaches in HCC.

Trial Name and ID	Cancer Type	Estimated Enrollment	Targeting Mechanism	Control Arm	Phase	Start and Completion Dates	Primary Measures
RATIONALE—301 [64] NCT03412773	HCC	December 2017	Tislelizumab (anti-PD-1 antibody)	Sorafenib	III	December 2017 July 2023	OS
Checkmate 9DW [65] NCT04039607	HCC	September 2019	Nivolumab + Ipilimumab	Sorafenib or lenvatinib	III	September 2019 June 2025	OS
NCT03764293 [45] (CARES-310)	Locally advanced or metastatic and unresectable HCC	June 2019	Camrelizumab (anti-PD-1 antibody) + Apatinib (VEGF inhibitor)	Sorafenib	III	June 2019 April 2023	OS PFS
DEDUCTIVE [62] NCT03970616	Advanced HCC	September 2019	Tivozanib (selective VEGFR 1,2,3 TKI) + Durvalumab (PD-L1 inhibitor)	N/A	1/IIb	September 2019 March 2023	TEAEs
NCT04183088 [63]	Advanced HCC	December 2020	Tislelizumab (anti-PD-1 antibody) + regorafenib (TKI)	N/A	II	December 2020 March 2025	TRAE ORR PFS
NCT04401813 [66]	Advanced HCC	June 2020	IBI308 (anti-CTLA4 antibody) + Sintilizumab (anti-PD-1 antibody)	N/A	I	June 2020 April 2023	AE ORR
NCT04212221 [67]	Advanced HCC	April 2020	MGD013 (anti PD 1 antiobdy and anti-LAG-3 antibody) + Brivanib	N/A	I/II	Completed Pending results	DLTs ORR
NCT03680508 [68]	Advanced HCC	December 2019	Cobolimab (TIM-3 binding antibody) + Dostarlimab (anti PD-1 antibody)	N/A	II	December 2019 October 2025	ORR
NCT03841201 [69]	Advanced HCC	June 2019	Lenvatinib (TKI) + Nivolumab (anti-PD-1 antibody)	N/A	II	June 2019 March 2023	ORR AE SAE
RENOBATE [70] NCT04310709	Advanced HCC	June 2020	Regorafenib + Nivolumab			Completed Pending results	Response Rate
ARYA-1 [71] NCT04502082	Advanced HCC	April 2021	ET140203 autologous T cell product	N/A	I/II	April 2021 June 2024	Incidence of AE and severity rates of AE Incidence rates of DLT RP2D
TRIPLET [72] NCT05665348	HCC—Hepatocellular Carcinoma	September 2021	Atezolizumab (anti-PD-L1 antibody) + Bevacizumab (VEGF inhibitor) + Ipilimumab (anti-CTLA-4 antibody)	Atezolizumab + Bevacizumab	II/III	September 2021 April 2026	Objective response Overall survival
NCT05022927 [73]	Advanced HCC	June 2021	ERY974 + Tocilicumab + Atezolizumab + Bevacizumab	N/A	I	June 2021 September 2024	Incidence of treatment-emergent adverse events (TEAE)
The PNeoVCA Study [74] NCT05269381	Various advanced solid tumors including HCC	March 2022	Cyclophosphamide (alkylating agent) + Neoantigen vaccine (containing sargramostim (GM-CSF)) + Pembrolizumab (anti-PD-1 antibody)	N/A	I	March 2022 February 2025	Incidence of AE
RELATIVITY—106 [75] NCT05337137	Advanced HCC	April 2022	Relatinib + Nivolumab + Bevacizumab	Nivolumab + Bevacizumab	1/2	April 2022 March 2023	Incidence of DLT PFS

**Table 4:** Small molecules in clinical trials.

Agent	Target	Clinical Trial	Cancer Type	Primary Objective
CA-170 Trial IDs: CTRI/2017/12/011026 (phase II) CTRI/2020/07/026870 (phase IIb/III)	PD-L1	Phase II	Lymphoma	ORR: 30%
	PD-L1	Phase IIb/III	Non-squamous, non-small cell lung cancer	ORR: ongoing
INCB086550 Trial ID: NCT04629339	PD-L1	Phase II	Select solid tumors	ORR: ongoing
CCX559 Trial ID: ACTRN12621001342808	PD-L1	Phase I	Solid tumors	Safety

**Table 5:** Small molecules in preclinical studies.

Small-Molecule Inhibitors in Preclinical Studies		
Molecule	Immune Checkpoint	Pathways
SMI402 in tumor-bearing mice [91]	TIM-3	Inhibition of tumor growth by increasing CD8+ T cell infiltration at tumor site
“Compounds 8 and 9” [92]	B7-1, preventing interaction with CTLA-4	Lack of inhibition in a cell adhesion assay
Tubeimoside-1 (TBM-1) [89]	PD-L1	Lysosomal degradation of PD-L1 in cancer cells via mTOR inactivation.
